# ﻿First records of *Trichina* Meigen, *Euthyneura* Macquart and *Oedalea* Meigen (Diptera, Hybotidae) from North Africa, with descriptions of two new species

**DOI:** 10.3897/zookeys.1124.90077

**Published:** 2022-10-10

**Authors:** Laila Zouhair, Patrick Grootaert, Kawtar Kettani

**Affiliations:** 1 Laboratory Ecology, Systematics, and Conservation of Biodiversity (LESCB), URL-CNRST N°18, FS, Abdelmalek Essaadi University, Tetouan, Morocco Abdelmalek Essaadi University Tetouan Morocco; 2 Royal Belgian Institute of Natural Sciences, O.D. Phylogeny and Taxonomy, Entomology, Vautier street 29, B1000 Brussels, Belgium Royal Belgian Institute of Natural Sciences Brussels Belgium

**Keywords:** Hybotidae, Morocco, new records, new species, North Africa, Oedaleinae, Trichininae

## Abstract

*Trichina* Meigen, 1830, *Euthyneura* Macquart, 1836 and *Oedalea* Meigen, 1820 are reported for the first time in North Africa from northern Morocco, with seven species including two ones new to science, based on material collected at nine sites located in the Moroccan sector of the Mediterranean Intercontinental Biosphere reserve (MIBR). These new records represent also the first evidence of the occurrence of Trichininae and Oedaleinae subfamilies throughout North Africa and bring the total of Moroccan hybotid fauna to 51 species. The new species are described and illustrated. A key to Moroccan*Trichina* species is provided.

## ﻿Introduction

*Trichina* Meigen, *Euthyneura* Macquart and *Oedalea* Meigen genera were previously included within the Trichiniini and Oedaleini tribes, which were included for a long time beside the Ocydromiini tribe in Ocydromiinae subfamily in Chvála’ s (1983) classification. More recent classifications, such as Sinclair and Cumming (2006) and Wahlberg and Johanson (2018), now treat these three tribes as separate subfamilies within the Hybotidae.

The genus *Trichina* attributed to the subfamily Trichininae consists of small black species (2.5–3 mm), with small mouthparts, large wing stigma extending to tip of R2+3, and hind tibiae dilated towards tip, and whose adults are known as predaceous in both sexes and occur for a long period during the summer in somewhat shaded humid biotopes (Chvála 1983). They are often found in the low grasses in deciduous forests, and are also observed at the tips of branches or along forest margins from approximately sunset until dusk (Chvála 1983). Larvae of *Trichina* occur probably in the soil (Smith 1989). The genera *Euthyneura* and *Oedalea* are both assigned to the Oedaleinae subfamily historically considered as Oedaleini tribe, which includes the most abundant of all flower-visiting empidoids in the Holarctic region (Marshall 2012). Species of *Euthyneura* are small black flies (2–3 mm) with a relatively long proboscis directed forward and simple legs devoid of long bristles; they are usually found on flowers and low herbage, but also on conifers (Chvála 1983). Among the representatives of the family Hybotidae, this genus represents one of the few groups whose adults feed only on nectar and pollen and are entirely flower visitors (Chvála 1983; Chandler 1992; Shamshev and Kustov 2012), while their larvae develop in rotting wood (Chvála 1983). The *Oedalea* species are more robust than those of *Euthyneura*. These are predaceous flies with conspicuously long antennae, shiny black thorax and raptorial hind legs (Chvála 1983; Shamshev 2020) and whose adults can usually be swept in low numbers from leaves of trees and shrubs, while the larvae can be bred from dead wood (Beuk 1992).

Various studies have been recently carried out on the aforementioned genera, mainly in Europe in the west Palaearctic region (Kanavalová et al. 2021). Thus, *Euthyneura* is known from the Palaearctic region with seven species (Shamshev and Kustov 2012) whereas data for *Trichina* and *Oedalea* in this region remain very poor and do not exceed seven species for *Trichina* and fourteen for *Oedalea* known to date from Europe (Barták and Kubík 2009; De Jong et al. 2014; Evenhuis and Pape 2021). As for North Africa, studies on hybotids are even scarcer and rather fragmentary, as is the case for Morocco where only 44 species of Hybotidae are recorded so far (Kettani et al. 2022), reflecting the lack of studies on such an important family of Diptera in the region.

In the present paper, we report the first record of *Trichina*, *Euthyneura* and *Oedalea* genera from the whole of North Africa, thus representing the first evidence of the occurrence of Trichininae and Oedaleinae subfamilies in North Africa, knowing that all species found so far in the west Palaearctic region of the genera of these subfamilies have only been recorded in Europe (De Jong et al. 2014), and no records have been reported until now in North Africa. Importantly, the genus *Euthyneura* is recorded here for the first time in the entire Mediterranean region.

In addition to these first records, we provide evenly here diagnosis and descriptions of two species described as new to science, belonging to the genus *Trichina*: *Trichinaazizi* Zouhair & Grootaert sp. nov. and *Trichinarifensis* Zouhair & Grootaert sp. nov.

## ﻿Materials and methods

The studied specimens originate from the entomological field surveys undertaken by the first author (LZ) over the years 2020 and 2021, and by the third author (KK) between 2017 and 2019. Specimens are preserved in 70° alcohol and housed in the private collection of the first author (PCLZ) at the University Abdelmalek Essaadi (Tetouan, Morocco) and in the private collection of the second author (PCPG). Most of the collected specimens were sampled using sweep net, and some with Malaise trap.

**Figure 1.**
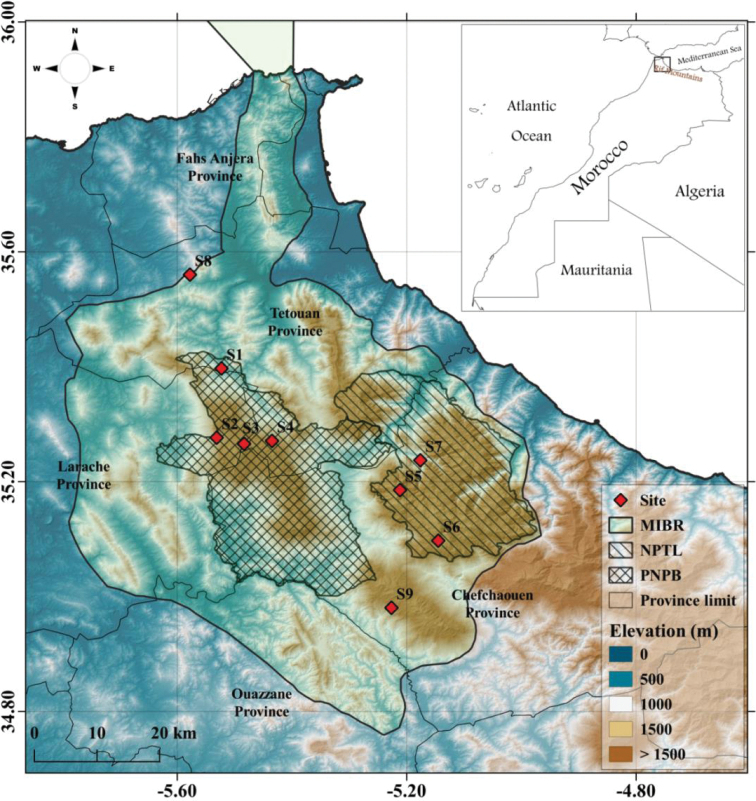
Location of the nine studied sites in the north of Morocco.

The specimens examined were mainly collected in mixed forests and riparian areas at nine sites located in the northern part of Morocco (Table 1, Fig. 1). With the exception of two forest sites situated within unprotected areas (S8, S9), the remaining sampling sites belong to the National Park of Talassemtane (**NPTL**) and the Project of Natural Park of Bouhachem (**PNPB**), which are considered the most important protected areas in terms of conservation in Morocco. The nine sites are located in the Rif, which consists of a mountainous chain located in the northernmost part of Morocco. This chain represents, along with the Atlas mountain region, the most important endemic areas of the country. It is important to highlight that all the studied sites belong also to the Mediterranean Intercontinental Biosphere Reserve (**MIBR**) which encompasses southern Spain and northern Morocco, and is known as a reservoir of biodiversity with regard to the Moroccan part (Rosas et al. 2017; Bachar et al. 2021).

**Table 1.** Coordinates, altitudes and localities of the studied sites.

Code	Site	Protected area, locality	Province	Altitude	Geographical coordinates	Habitat	Collecting tool
S1	Tissgris	PNPB, Hmmadesh	Tetouan	505 m	35.397°N, 5.522973°W	Mixed forest	Sweep net
S2	Oued Stah	PNPB, Bni Aarouss	Larache	766 m	35.2764°N, 5.531389°W	River bank	Sweep net
S3	Oued Asellam	PNPB, Moulay Abdessalam	Tetouan	1267 m	35.2657°N, 5.483595°W	River bank	Sweep net
S4	Lemtahane	PNPB, Dar Abdessalam	Tetouan	964 m	35.2708°N, 5.434864°W	Mixed forest	Malaise trap
S5	Sefihat telj	NPTL, Talembote	Chefchaouen	1745 m	35.1852°N, 5.211767°W	Fir forest	Sweep net
S6	Bouslimane	NPTL, Jbel Bouslimane	Chefchaouen	1350 m	35.0971°N, 5.14505°W	Fir forest	Sweep net
S7	Oued El Ferda	NPTL, Akoumi	Chefchaouen	447 m	35.237°N, 5.176283°W	River bank	Sweep net
S8	Aïn Lahcen	Unprotected area, Aïn Lahcen	Tetouan	316 m	35.5601°N, 5.578017°W	Pine forest	Sweep net
S9	Fifi	Unprotected area, Bab Taza	Chefchaouen	1332 m	34.9803°N, 5.2266°W	Mixed forest	Sweep net

In the Rif, the climate is of the Mediterranean type and is composed of two distinct periods, a hot and dry summer and the other a relatively cold and rainy winter (Al Karkouri 2017). The bioclimate is primarily wet with high humidity which allows the growth of a rich flora, including nearly all of the Moroccan forest species such as deciduous groves, the endemic fir forests of Morocco (*Abiesmarocana*), the Atlas Cedar tree (*Cedrusatlantica*), Pine forests (*Pinuspinaster*), Cork Oak (*Quercussuber*), Olives, Thuja articulata (*Tetraclinisarticulata*), Kermes Oak (*Quercuscoccifera*), Holm Oak (*Quercusilex*), and the coccifera which have formed extensive forests in the western part of the Rif (Taiqui 1997).

Most species were recognized using a stereomicroscope, but for the new ones it was necessary to make a preparation of the male genitalia for their accurate identiﬁcation. The male terminalia were removed from the insect body, macerated in the 10% KOH for 24h in order to dissolving the tissues. When all darkly sclerotized structures were transparent enough, the maceration process was stopped by removing the terminalia from the KOH and bringing them to a vial with tap water. After at least 5 minutes a drop of vinegar was added to the vial to stop the maceration process completely. After about a minute the terminalia were rinsed in vial with tap water again and after a few minutes transferred to 70% ethanol for 10 minutes and transferred to pure glycerin. They were preserved in the glycerol to be later observed under the microscope and drawn using a camera obscura.

Morphological terminology and abbreviations largely follow Chvála (1983), except for the male terminology, which mostly follows Sinclair and Cumming (2006). The arrangement of taxa follows also Sinclair and Cumming (2006).

## ﻿Results

### ﻿Family HYBOTIDAE Meigen, 1820

**Subfamily TRICHININAE Chvála, 1983**

#### Genus *Trichina* Meigen, 1830

##### ﻿ Trichina elongata

Taxon classificationAnimaliaDipteraHybotidae

Haliday, 1833

1C6ADAA9-1822-5597-BFF3-D4BAAFC58272

Fig. 7A

###### Material examined.

1♂. Morocco, Rif, Sefihat telj, NPTL, 1745 m, 04.iii.2020, sweep net, leg. L. Zouhair, PCLZ; 1♂. Rif, Akoumi, Oued El Ferda, NPTL, 447 m, 13.iii.2021, sweep net, leg. L. Zouhair, PCLZ.

###### Distribution.

Widespread throughout Europe, including the European part of Russia (Shamshev and Kustov 2006). First record for Morocco.

##### ﻿ Trichina opaca

Taxon classificationAnimaliaDipteraHybotidae

Loew, 1864

8625C6ED-092C-5A8C-AC0F-D1BDAB45CFB6

Fig. 7B

###### Material examined.

1♂. Morocco, Rif, Fifi, 1332 m, 6.v.2021, sweep net, leg. L. Zouhair, PCLZ.

###### Distribution.

Known from Central and Northern Europe (Shamshev and Kustov 2006). First record for Morocco.

##### ﻿ Trichina unilobata

Taxon classificationAnimaliaDipteraHybotidae

Chvála, 1981

9D6310A2-528E-51EA-9EAD-86C4744DCFFD

Fig. 7C

###### Material examined.

1♂. Morocco, Rif, Aïn Lahcen, 316 m, 10.i.2020, sweep net, leg. L. Zouhair, PCLZ.

###### Distribution.

Spain, Turkey. First record for Morocco.

### ﻿Descriptions of new species

#### ﻿ Trichina azizi

Taxon classificationAnimaliaDipteraHybotidae

Zouhair & Grootaert sp. nov.

D49CFADD-FC3B-5A6F-A30C-CF1DCC4F5E9A

https://zoobank.org/E397A731-4350-4B07-8EA1-16E81947642E

Figs 3A–D , 7D

##### Material examined.

***Holotype*.** 1♂. Morocco, Rif, Bouslimane, NPTL, 1350 m, 28.iv.2019, sweep net, leg. K. Kettani, PCLZ.

##### Habitat.

**Figure 2.**
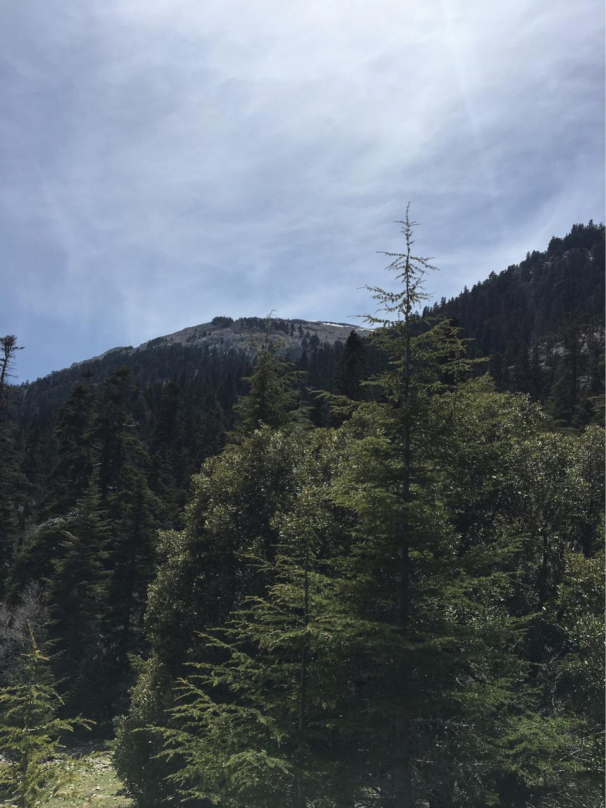
Type habitat of *Trichinaazizi* Zouhair & Grootaert, sp. nov. in the fir forest at Bouslimane locality (Photo: K. Kettani, 28.04.2019).

(S6: Bouslimane) (Fig. 2): The holotype was swept from the foothills of Bouslimane mountain located at the south of the national park of Talassemtane. The habitat consists of a fir forest (*Abiesmarocana*) dominated by a humid bioclimate as part of the mesomediterranean zone. This fir formation grows on brown fersiallitic soil.

##### Differential diagnosis.

*Trichinaazizi* sp. nov. has 3^rd^ antennal segment considerably long and slender like in *T.elongata* according to the specimen we have and to the description in Chvála (1983), but it differs in length since it is less than 3.4 times as long as broad in *T.elongata* (Barták and Kubík 2009) whereas it is more than 3.6 times as long as broad in the new species. Both species differ also in the length of the stylus, which in the new species is little longer than half-length of 3^rd^ segment; while in *T.elongata*, the stylus is less than half the length of the 3^rd^ segment. The bare terminal part of antennae is shorter than the pubescent thicker basal part which is the same in both. The new species has only two pairs of scutellar setae, like in *T.elongata* and also in *T.clavipes* and *T.bilobata*. In addition, it can be recognized by yellowish hind trochanter, femora, tibiae and metatarsi, and by the lack of a ventral spine on hind trochanters, like in *T.opaca*. Male terminalia with the hypandrial projection is rather long, like in *T.elongata*, but in the latter it is more slender than in the new species. The left surstylus is similar in structure to that of *T.elongata*, but it is not sharpened apically like in *T.elongata*. The right surstylus is spiny apically and sub-apically, which is the most important diagnostic character in this new species. Cerci are long and well developed but the left cercus is longer than the right one.

##### Etymology.

This species is dedicated to the father (Aziz Zouhair) of the first author.

##### Description.

**Male**. Small brown species (2.8 mm) (Fig. 7D).

***Head*.** Brown in ground colour. Ommatidia bicoloured. Antennae brown, with postpedicel considerably long and slender with 0.22 mm long over 0.06 mm wide, stylus is 0.134 mm long (0.6 × the length of postpedicel hence a little longer than half the length), its bare terminal part shorter than the pubescent thicker basal part. Palpus small, brownish, with a brownish bristle. Proboscis paler. Face narrow, less than width of scape.

***Thorax*.** Entirely brown, (unfortunately, all thoracic setae are broken, but according to setae follicles, 2 pairs of scutellar setae). Mesonotum shining black except for the extreme anterior border that bears some sparse microtrichia, the notopleural depression with some denser microtrichia, but is still subshining black. Wings very faintly brown yellowish infuscated, veins pale brown, pterostigma yellowish brown and indistinct, costa running to tip of M1, squamae including fringes brown, halters brown yellowish. Legs almost uniformly brownish leaving hind trochanter, femora, tibia, and hind metatarsi yellowish. Fore femur slender with paler hairs; mid femur with 2 ventral rows of dark and thick bristles; hind femur longer than mid and fore femora, with a row of thick and long spine-like bristles. No spine on the hind trochanter. Fore tibia more slender at base, covered with small paler hairs; mid tibia very slender than fore tibia and covered mostly with dark hairs; hind tibia much longer than fore and mid tibiae, laterally compressed and dilated towards tip.

**Figure 3.**
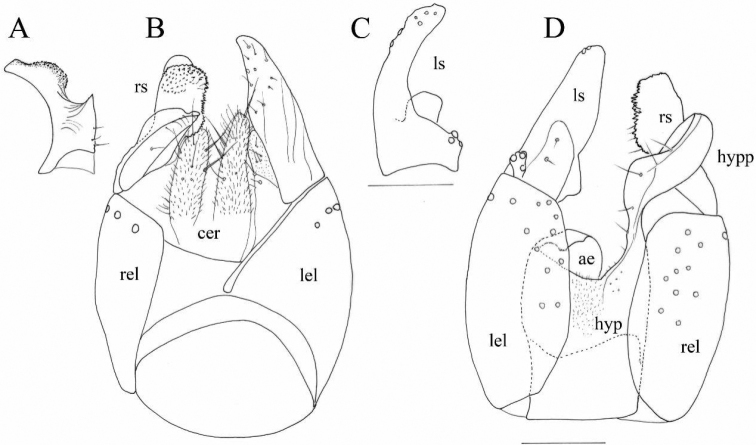
*Trichinaazizi* Zouhair & Grootaert sp. nov., Holotype male, terminalia **A** right surstylus, lateral view **B** epandrium, dorsal view **C** left surstylus, lateral view **D** epandrium, ventral view. Scale: 0.1 mm. (Drawn by Patrick Grootaert).

***Abdomen*.** Brown, tergites and sternites covered with scattered black setulae. Terminalia somewhat large and blackish brown, with spiny right surstylus in apical and sub-apical parts (Fig. 3B, D), left surstylus longer and slender in comparison with right surstylus, hypandrial projection moderately long, slender (Fig. 3D). Cerci longer than wider, the left cercus longer than the right cercus (Fig. 3B).

**Female.** (Unknown).

Abbreviations: ae: aedeagus (phallus); cer: cercus; hyp: hypandrium; hypp: hypandrial projection; lel: left epandrial lamella; ls: left surstylus; rel: right epandrial lamella; rs: right surstylus.

#### ﻿ Trichina rifensis

Taxon classificationAnimaliaDipteraHybotidae

Zouhair & Grootaert sp. nov.

1DDE80D4-323B-5F21-807C-6EE93BA65B29

https://zoobank.org/FB3A6D61-8C16-4AAC-A20D-E7D7EA783F7A

Figs 5A–D , 7E

##### Material examined.

***Holotype*.** 1♂. Morocco, Rif, PNPB, Lemtahane, 964 m, 7.v.2017–30.v.2017, Malaise trap, leg. K. Kettani, PCLZ.

##### Habitat.

**Figure 4.**
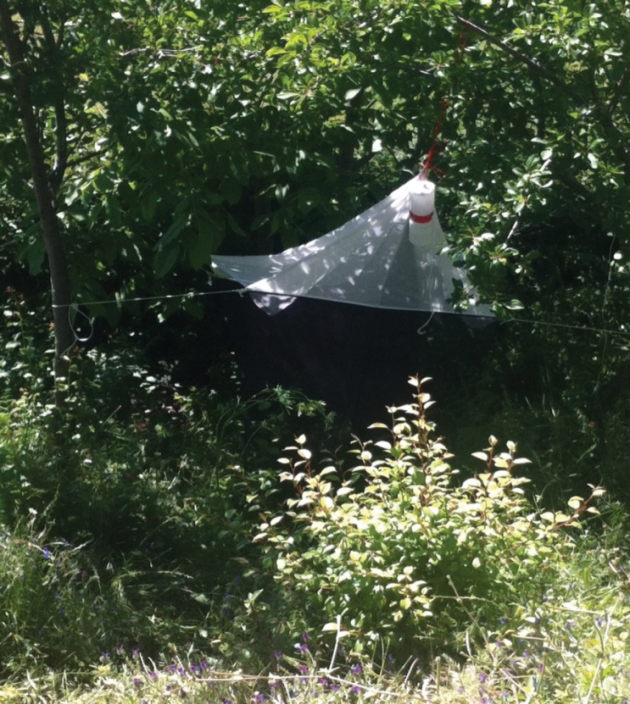
Type habitat of *Trichinarifensis* Zouhair & Grootaert sp. nov., in Lemtahane (Photo: K. Kettani, 07.05.2017).

(S4: Lemtahane) (Fig. 4): The Malaise trap was set up in a scrubland composed of *Pinuspinaster* and some fruit trees growing on siliceous soil. The bioclimate is subhumid and favours thermo-Mediterranean vegetation.

##### Differential diagnosis.

The newly described species *Trichinarifensis* sp. nov., is very similar to *T.opaca* as it is described in Chvála (1983) and according to the key of *Trichina* in Barták and Kubík (2009) and to our *T.opaca* specimen, in: the absence of the ventral spine in hind trochanters, and in the colour of legs which are extensively blackish to blackish brown, with the knees and extreme base of tibiae often paler. It is similar to *T.opaca* also in having the same number of scutellar pairs setae (3 pairs). The wings are conspicuously darkened brown, veins blackish, squamae including fringes and halters deep black in both species. However, both species differ in the pruinosity of the mesonotum: in the new species, the mesonotum is shining black but anteriorly there is a very narrow stripe with weak microtrichia and the notopleural depression is more distinctly set with microtrichia (not grey dusted), while in *T.opaca* the mesonotum is entirely thinly brownish pollinose and consequently rather dulled, and differ also in the length of the 3d antennal segment that is less than 3.2 times as long as broad in *T.opaca*, while it is more than 3.2 times (4 times) as long as broad in *T.rifensis* sp. nov. The male terminalia in the new species is with the hypandrial projection much longer than in *T.opaca*, the structure of the left surstylus is similar in the two species so that is C-shaped in both. The right surstylus of the new species is spiny in the apical and sub-apical parts contrary to *T.opaca*, which forms an important differential diagnosis character, cerci are equal in length and shape in *T.opaca*, while in the new species, the left cercus is narrower and longer than the right cercus which is shorter and conical in shape.

##### Etymology.

The new species is named *rifensis*, after the Moroccan Rif region where it was found.

##### Description.

**Male**. Black species with body small (3 mm) Fig. 7E.

***Head*.** Black in ground colour. Eyes meeting on frons for a long distance. A distinct prominent ocellar tubercle with 2 pairs of ocellar bristles, anterior pair as long as posterior one, occiput and vertex finely greyish pollinose, covered with black and distinct hairs. Face linear, narrow less than width of scape. Antennae entirely black, inserted at middle of head in profile with postpedicel is 0.24 mm long over 0.06 mm wide, stylus (apical naked part missing) has the basal part and the thickened second segment 0.079 mm long. Palpus short with a very long black apical bristle, 1.5 times as long as palpus and a subapical bristle half as long as apical one. Proboscis yellowish, pointing obliquely forward and covered with several brownish hairs.

***Thorax*.** Polished black with all hairs black and long. Acr biserial, dc uniserial, ending in 1 pair of very long prescutellars, 1 humeral, 1 notopleural, 1 postalar bristles, 3 scutellar pairs. Mesonotum covered with microtrichia. Mesonotum shining black but anterior part with a very narrow stripe with weak microtrichia, notopleural depression more distinctly set with microtrichia (not grey dusted). Wings conspicuously brownish, stigma blackish brown extending to tip of vein R2+3, veins blackish brown, costa reaching vein M1, squamae, including fringes, and halters black. Legs mostly covered with hairs, extensively blackish leaving base of hind tibia and knees paler. No spine on hind trochanter. Fore femur rather slender, covered with paler hairs and bearing 6–8 black bristles apically; mid femur with 2 rows of paler bristles and 2–4 black bristles apically; hind femur much longer than fore and mid femora with ventral and dorsal paler bristles. Fore tibia rather slender, with black hairs; mid tibia with distinct a pair of black and strong bristles; hind tibia much longer than fore and mid tibiae, laterally compressed and dilated towards tip, with 2 rows of black bristles, with one dorsal black bristle.

**Figure 5.**
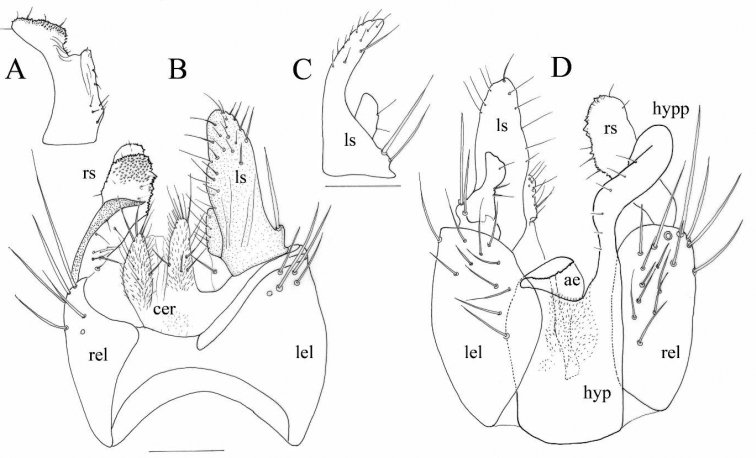
*Trichinarifensis* Zouhair & Grootaert sp. nov., Holotype male terminalia **A** right surstylus, lateral view **B** epandrium, dorsal view **C** left surstylus, lateral view **D** epandrium, ventral view. Scale: 0.1 mm. (Drawn by Patrick Grootaert).

***Abdomen*.** Tergites and sternites blackish, covered with scattered black setulae. Terminalia black, with right surstylus somewhat robust and spiny in sub-apical and apical parts (Fig. 5B, D), left surstylus slender and hypandrial projection long and slender. Right cercus shorter, conical, left cercus longer and narrower (Fig. 5B).

**Female.** (Unknown)

Abbreviations: ae: aedeagus (phallus); cer: cercus; hyp: hypandrium; hypp: hypandrial projection; lel: left epandrial lamella; ls: left surstylus; rel: right epandrial lamella. rs: right surstylus.

##### General comments.

Despite the male terminalia of the two new species described above being very similar in structure, it is easy to distinguish between the both species: *Trichinaazizi* sp. nov. has yellowish hind trochanters, femora tibiae and metatarsi, so it is very distinct from *Trichinarifensis* sp. nov., which has entirely black hind trochanters, femora, tibiae (except at base) and tarsi, as may be seen in Fig. 7D, E.

Compared to other species of *Trichina* described so far, *Trichinarifensis* sp. nov., is very close to *Trichinaopaca* in most morphological characters (colour of legs and wings, shape of antennae, number of scutellar pairs, absence of hind trochanters spine(s)) but as we previously noted in the differential diagnosis to this new species, it differs clearly in the male terminalia. As to *Trichinaazizi* sp. nov., is similar to *T.elongata* in the shape of the antennae, number of scutellar pairs and absence of the trochanter spine, but it is easy to differentiate these species by the male terminalia as we have described in the differential diagnosis above. The common character between the right surstylus of the both new species is that they are spiny, which is a character not described in *Trichina* at all.

### ﻿Key to the *Moroccan* species of *Trichina* Meigen (males)

This key is compiled by referring to the key of *Trichina* in Barták & Kubík (2009).

**Table:** 

1	The whole of mesoscutum microtrichose. 3d antennal segment usually less than 3.2 times as long as broad	***T.opaca* Loew, 1864**
–	The central parts of mesoscutum without microtrichiae. 3d antennal segment more than 3.4 times as long as broad	**2**
2	Face broader	***T.unilobata* Chvála, 1983**
–	Face narrow	**3**
3	Hind trochanter with posterior to ventral spine(s)-like setae. Legs uniformly brownish. Right surstylus not spiny in apical and sub-apical parts	***T.elongata* Haliday, 1833**
–	Hind trochanter without posterior to ventral spine(s)-like setae. Hind trochanter, femora, tibia and metatarsi yellowish (*T.azizi* sp. nov.) or at least knees and base of hind tibia paler (*T.rifensis* sp. nov.). Right surstylus spiny in apical and sub-apical parts	**4**
4	3 pairs of scutellar setae	***T.rifensis* sp. nov.**
–	2 pairs of scutellar setae	***T.azizi* sp. nov.**

### ﻿Subfamily OEDALEINAE Chvála, 1983

#### Genus *Euthyneura* Macquart, 1836

##### ﻿ Euthyneura myrtilli

Taxon classificationAnimaliaDipteraHybotidae

Macquart, 1836

4A9ACA37-2B45-5851-918A-8CF49B3CDF0B

Fig. 7F

###### Material examined.

1♀. Morocco, Rif, PNPB, Lemtahane, 964 m, 7.v.2017–30.v.2017, Malaise trap, leg. K. Kettani, PCLZ; 1♀. Rif, PNPB, Oued Asellam, 1267 m, 22.iii.2021, sweep net, leg. L. Zouhair, PCLZ ; 1♀. Rif, PNPB, Oued Stah, 766 m, 2.v.2021, sweep net, leg. L. Zouhair, PCLZ.

###### Distribution.

Common in northern and central Europe, absent in the south (Chvála and Vonicka 2008); also European part and Western Siberia of Russia (Shamshev 2016). First record for Morocco.

#### Genus *Oedalea* Meigen, 1820

##### ﻿ Oedalea portugalica

Taxon classificationAnimaliaDipteraHybotidae

Barták & Grootaert, 2021

2203FB17-A055-57FC-A975-EF0B123A5478

Figs 6A–E , 7G

###### Material examined.

1♀. Morocco, Rif, PNPB, Tissegris, 505 m, 20.iv.2021, sweep net, leg. L. Zouhair, PCLZ.

###### Distribution.

Known up to present only from the type locality in Portugal. First record for Morocco.

###### Remarks.

*Oedaleaportugalica* was described by Barták & Grootaert (2021) in Kanavalová et al. (2021) from Portugal. The male terminalia were not illustrated or described, and are provided herein for the first time.

**Figure 6.**
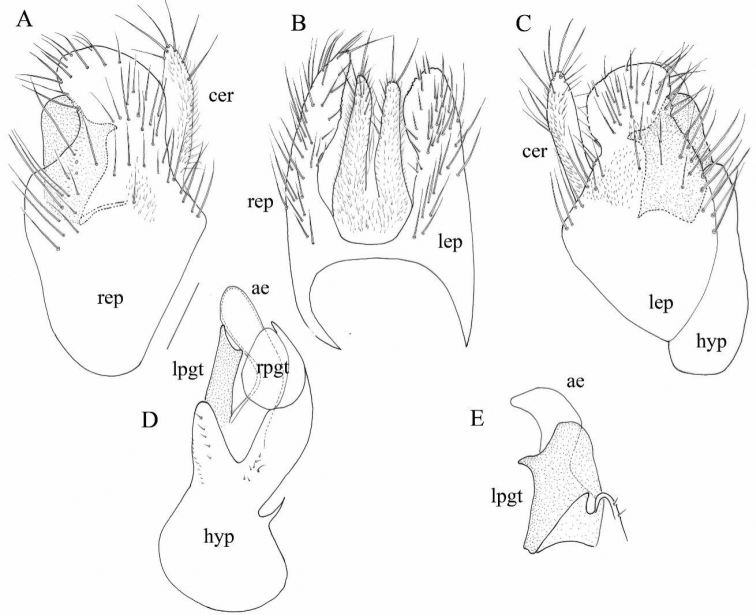
*Oedaleaportugalica* Barták & Grootaert, 2021. Male terminalia **A** right epandrial lamella, lateral view **B** epandrium, dorsal view **C** left epandrial lamella, lateral view **D** hypandrium, ventral view **E** left postgonite with aedeagus (phallus). Scale: 0.1 mm. (Drawn by Patrick Grootaert).

Terminalia (Fig. 6) small. Cerci (Fig. 6B) digitiform, covered toward tip with long setae, right cercus (Fig. 6B) slightly longer and more narrowed apically than left cercus. Epandrium covered with long setae (Fig. 6A, C), right epandrial lamella somewhat longer, broader and more elongated towards apex (Fig. 6A) than left epandrial lamella (Fig. 6C).

In Kanavalová et al. (2021), *O.portugalica* was compared with *O.stigmatella*. In the terminalia of *O.stigmatella* (figs 401–404 in Chvála 1983), the shape of the right and left epandrial lamellae is different, so that in *O.portugalica* there is a deep medial apical incision in the hypandrium (it is forked Fig. 6D), while in *O.stigmatella* the fork is shallow and broader (fig. 403 in Chvála 1983). The tip of the left postgonite is very characteristic and different. A sharp protrusion is apical in *O.stigmatella* (fig. 404 in Chvála 1983) while it is lateral (subapical) in *O.portugalica* (Fig. 6E seen from the right, while on Fig. 6A it is below the right epandrial lamella seen from the right side).

Abbreviations: ae: aedeagus (phallus); cer: cercus; hyp: hypandrium; lep: left epandrial lamella; lpgt: left postgonite; rep: right epandrial lamella; rpgt: right postgonite.

**Figure 7.**
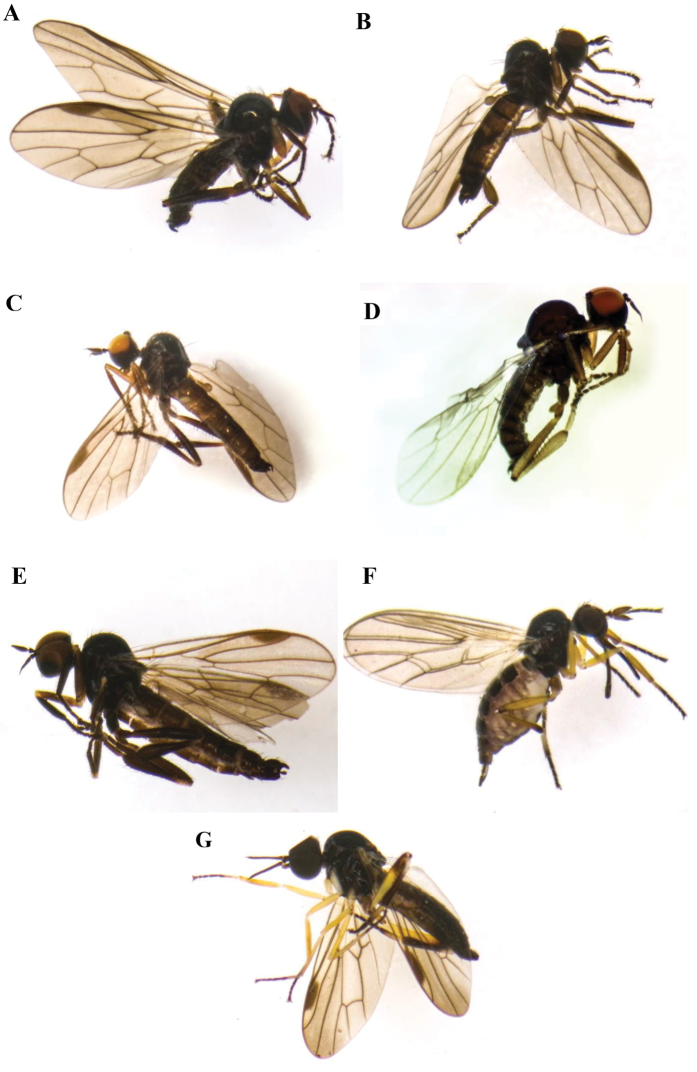
**A***Trichinaelongata* Haliday, 1833 **B***Trichinaopaca* Loew, 1864 **C***Trichinaunilobata* Chvála, 1981 **D***Trichinaazizi* Zouhair & Grootaert sp. nov. **E***Trichinarifensis* Zouhair & Grootaert sp. nov. (Left antennal style missing) **F***Euthyneuramyrtilli* Macquart, 1836 **G***Oedaleaportugalica* Barták & Grootaert, 2021.

## ﻿Discussion

The current study contributes significantly to the enrichment of the faunistic database of the Hybotidae fauna of Morocco in particular and that of North Africa in general. Seven species belonging to three genera (*Trichina*, *Euthyneura* and *Oedalea*) which were unprecedentedly reported in North Africa as well as their respective subfamilies (Trichininae and Oedaleinae) are newly recorded in the region, increasing the total of the known hybotid species of Morocco from 44 species to 51. The description of two new species belonging to the *Trichina* genus provides also an important contribution to the hybotid fauna. Our results show that the distribution of the three mentioned genera has expanded beyond Europe where they were originally recorded.

These findings highlight the richness of the Moroccan biodiversity, particularly in the Western Rif, which is considered a biodiversity hotspot, where the new species were found, and which constitutes the only part of Morocco included in the Mediterranean Intercontinental Biosphere Reserve (MIBR) due to the great specific richness and the high rate of endemism recorded there (Bachar et al. 2021). Indeed, of the 51 species of Hybotidae occurring in Morocco, 25 of them are cited exclusively in the Rif.

As noted by Stark (2008) regarding the weak diversity of the subfamilies Trichininae and Oedaleinae compared to the Tachydromiinae, our results exhibit the same pattern following our study of ancient and recent hybotid samples collected from various habitats in Morocco by the first and the third authors, where Tachydromiinae subfamily was remarkably always dominant. However, this can also be explained by the fact that the MoroccanHybotidae fauna is strongly under-collected and there is still at least as much to discover there. There is no doubt that the number of species of Hybotidae occurring in Morocco as in North Africa will increase when collecting is more intensified.

## Supplementary Material

XML Treatment for Trichina elongata

XML Treatment for Trichina opaca

XML Treatment for Trichina unilobata

XML Treatment for Trichina azizi

XML Treatment for Trichina rifensis

XML Treatment for Euthyneura myrtilli

XML Treatment for Oedalea portugalica
